# *Exocarpium Citri Grandis* Ameliorates Metabolic Disorders by Inhibiting Inflammatory Responses and NLRP3 Inflammasome Activation

**DOI:** 10.1002/fsn3.71074

**Published:** 2025-10-28

**Authors:** Tingting Chen, Xiaoqin Wu, Danna Wang, Tianqi Cui, Jiayu Li, Ziyi Zhang, Min Qiu, Chenlu Ma, Hengrui Liu, Yong Gao, Jiangtao Zeng, Hang Li, Jiawen Huang

**Affiliations:** ^1^ State Key Laboratory of Traditional Chinese Medicine Syndrome, Science and Technology Innovation Center Guangzhou University of Chinese Meidicne Guangzhou China; ^2^ Cancer Research Institute Jinan University Guangzhou China; ^3^ Guangzhou University of Chinese Medicine Maoming Hospital (Maoming Hospital of Traditional Chinese Medicine) Maoming China; ^4^ Central lab, Shenzhen Bao'an Chinese Medicine Hospital, Guangzhou University of Chinese Medicine Shenzhen China

**Keywords:** AKT, ECG, metabolic syndrome, molecular dynamics, ovariectomized, PI3K

## Abstract

*Exocarpium Citri Grandis* (ECG, known as *Huajuhong* in Chinese), as the fruit that can be used both as medicine and food, has gained attention for its hypolipidemic and anti‐obesity effects. However, its lipid‐lowering potential and ability to alleviate metabolic syndrome (MS) remain unexplored. This study aimed to confirm the therapeutic effects of ECG on postmenopausal MS and investigate its underlying mechanisms. An animal model of female postmenopausal MS was established using 8‐week‐old ovariectomized (OVX) mice. Starting from the third week after model establishment, the mice were orally administered at the doses of 25, 50, and 100 mg/kg for 12 consecutive weeks. HepG2 cells co‐induced by oleic acid and palmitic acid (OA&PA) were used as an in vitro model. Ultra‐high‐performance liquid chromatography‐Q‐Exactive (UHPLC‐Q‐Exactive) analysis, histological examination, immunofluorescence, network pharmacology, molecular docking, molecular dynamics simulations, and other methods were employed for further analysis. ECG contains ingredients such as naringin and narirutin. In vitro experiments showed that ECG intervention significantly reduced reactive oxygen species (ROS) accumulation and downregulated the mRNA expression of *Srebp1, G6, Fas, Pgc1α, Acc, Casp1, Nfkb, IL1β, IL6, Txnip, Nlrp3*, and *ASC*. Additionally, ECG inhibited the protein expression of NF‐κB, Txnip, NLRP3, ASC, and Caspase‐1, while enhancing the expression of Trx‐1. In vivo, ECG alleviated metabolic disorders in OVX mice by reducing lipid accumulation, attenuating inflammatory responses, and inhibiting NLRP3 inflammasome activation. Network pharmacology analysis and molecular dynamics identified the PI3K/AKT1 signaling pathway as a crucial target mediating the functional effects of ECG. ECG exerts therapeutic benefits in ameliorating postmenopausal MS‐related metabolic disorders by inhibiting inflammatory responses, suppressing NLRP3 inflammasome activation, and modulating the PI3K/AKT1 signaling pathway, providing new insights into the application of ECG for managing postmenopausal MS.

## Graphical abstract

Exocarpium Citri Grandis (ECG, Huajuhong in Chinese), as the fruit of a plant that can be used both as medicine and food, has gained attention for hypolipidemic and anti‐obesity effects. To our knowledge, this is an innovative study based on in vivo and in vitro experiments aimed at exploring the pharmacological mechanisms of ECG in ameliorating MS after menopause. Our findings provide new insights into the application of foods that are homologous to medicine and food in managing menopausal MS.

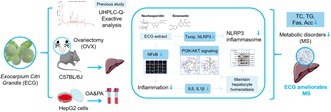

**Abbreviations**
ALD: Alcoholic liver disease
ALT: Alanine aminotransferase
AST: Aspartate transaminase
BCA: Bicinchonininc acid
CCK8: Cell Counting Kit‐8
E2: Estradiol
ECG: Exocarpium Citri Grandis
ECL: Enhanced chemiluminescence
GO: Gene ontology
H&E: Hematoxylin and eosin
HRT: Hormone replacement therapy
KEGG: Kyoto encyclopedia of genes and genomes
MAFLD: Metabolic Dysfunction‐associated Fatty Liver Disease
MD: Molecular dynamics
MS: Metabolic syndrome
OA: Oleic acid
OCT: Optimal cutting temperature
ORO: Oil red O
OVX: Ovariectomized
PA: Palmitic acid
PPARG: Peroxisome proliferator‐activated receptor gamma
PPI: Protein–protein interaction
qPCR: Quantitative PCR
RMSD: Root mean square deviation
TC: Total cholesterol
TG: Triglycerides
WB: Western blot

## 1 Introduction

During menopause, the decline in ovarian function is accompanied by a relative increase in circulating androgens; this increase further disrupts lipid metabolism, alters the lipid profiles, and triggers a spectrum of endocrine and metabolic abnormalities (Ko and Kim 2020). When combined with modern lifestyle shifts and suboptimal dietary patterns, particularly higher intake of high‐fat diets, this hormonal change exacerbates metabolic dysregulation, which is an effect further compounded by impaired insulin signaling pathways and the upregulation of pro‐inflammatory cytokines (Mishra et al. 2021). In postmenopausal women, high‐fat diets not only exacerbate hepatic steatosis but also may accelerate the onset and progression of liver fibrosis (Ali et al. 2022; Sucedaram et al. 2021). Current interventions for metabolic abnormalities in postmenopausal women primarily center on lifestyle modifications and pharmacotherapies, with the latter including metformin and glucagon‐like peptide‐1 (GLP‐1) receptor agonists (Model et al. 2024). However, hormone replacement therapy (HRT) is associated with potential risks, such as a heightened risk of breast cancer and other malignancies (Davies and Hamoda 2019; Manson et al. 2024). As such, the development of new strategies for managing metabolic syndrome (MS) in postmenopausal women has become an area of growing focus.

Metabolic syndrome is a prevalent metabolic disorder closely associated with a high incidence of obesity, and its pathophysiological mechanisms predominantly involve insulin resistance, excessive release of free fatty acids, and particularly, a chronic proinflammatory state (Eckel et al. 2005). It has been demonstrated that hepatic insulin resistance induced by metabolic dysregulation is closely associated with increased expression and overproduction of inflammatory mediators, including TNFα, IL‐6, and IL‐1β (Cai et al. 2005). Notably, the liver is one of the primary organs involved in obesity‐related inflammation, while the proinflammatory cytokine IL‐1β contributes to the pathogenesis of MS through activating the NLRP3 inflammasome; in this process, the inflammasome facilitates the release of additional proinflammatory cytokines that exacerbate hepatic insulin resistance and dysregulated lipid metabolism (Esser et al. 2014). Accumulating evidence suggests that the NLRP3 inflammasome plays a central role in obesity‐induced insulin resistance (Esser et al. 2013; Litwiniuk et al. 2021; Vandanmagsar et al. 2011). Previous studies have highlighted the significant involvement of the PI3K/AKT1 signaling pathway in regulating inflammatory responses, as well as its upstream regulatory role in NLRP3 inflammasome activation (Xiong et al. 2023). Thus, alleviating the inflammatory response and targeting the NLRP3 inflammasome may serve as effective therapeutic strategies for the treatment of MS.

*Exocarpium Citri Grandis* (ECG), also known as “*Huajuhong*”, a traditional food‐medicine homologous plant of the *Citrus* family, is widely used in foods and health products. Research has shown that ECG offers multiple health benefits, including reducing inflammation and counteracting oxidative stress (Jiang et al. 2014). Our previous studies also identified the anti‐inflammation effect of ECG, with NFκB being a potential target (Xu et al. 2024). Currently, the active compounds in ECG have attracted significant attention, particularly for their potential in managing Metabolic dysfunction‐associated fatty liver disease (MAFLD), and this finding suggests that ECG may help restore metabolic balance (Deng et al. 2023). It has been demonstrated that the active ingredients of ECG can lower blood glucose and lipid levels, primarily by reducing oxidative stress, inhibiting digestive enzyme activity, and improving lipid metabolism (Ko and Kim 2020). However, there remains a lack of in‐depth research into the mechanisms by which ECG alleviates postmenopausal metabolic disorders, such as elevated blood glucose and lipid levels. In this study, in vivo and in vitro experiments were conducted to investigate the pharmacological effects and underlying mechanisms of ECG in alleviating postmenopausal MS, with the goal of providing a scientific basis for the application of ECG in metabolic health management.

## 2 Material and Methods

### 2.1 Reagents and Materials

Palmitic acid (PA) and oleic acid (OA) were purchased from Aladdin Scientific (CHN). Antibodies against AKT, phosphorylated AKT (p‐AKT), PI3K, phosphorylated PI3K (p‐PI3K), and PPARα were obtained from Abmart (CHN). Additionally, antibodies targeting Txnip, Trx‐1, NLRP3, ASC, NF‐κB p‐p65 (S536), and Caspase‐1 were purchased from Affinity Biosciences (CHN). The β‐ACTIN antibody was acquired from Servicebio (CHN).

### 2.2 UHPLC‐Q‐Exactive Analysis of ECG

The preparation and analysis of ECG were performed following previously established methodologies (Huang et al. 2024; Xu et al. 2024). The components of the ECG batch used in this study have been identified via UHPLC‐Q‐Exactive analysis in previously published reports, and it was mainly composed of Narirutin, Naringin, Neohesperidin et al. (Xu et al. 2024). Briefly, 100 mg of lyophilized ECG material was dissolved in 1 mL of methanol, followed by vortex mixing. Cryogenic homogenization was conducted at −40°C for 2 min at 60 Hz, after which ultrasonication was performed in an ice bath for 60 min. The mixture was then equilibrated at low temperature (−40°C for 30 min). For centrifugation, primary centrifugation was performed at 12,000 rpm and 4°C for 10 min; the supernatant was filtered, refrigerated at 4°C overnight, and subjected to secondary centrifugation. The resulting supernatant was diluted 10‐fold with methanol and filtered again prior to chromatographic analysis. Chromatographic separation was carried out using an ACQUITY UPLC HSS T3 column (100 × 2.1 mm, 1.8 μm), which was maintained at 45°C with a flow rate of 0.35 mL/min. The mobile phase consisted of 0.1% formic acid (phase A) and acetonitrile (phase B), with the following gradient program: 0–2 min (95% A), 2–4 min (95% → 70% A), 4–8 min (70% → 50% A), 8–10 min (50% → 20% A), 10–15 min (0% A), followed by 15.1–16 min re‐equilibration (re‐equilibration to 95% A). The injection volume was 5 μL, and detection was performed using a photodiode array (PDA) detector over the wavelength range of 210–400 nm. Mass spectrometric analysis was conducted using heated electrospray ionization with dual‐polarity scanning (positive and negative modes). Data acquisition was performed via data‐dependent analysis (DDA) in the Full MS/dd‐MS^2^ (Top 8) scanning mode.

### 2.3 Animals

Eight‐week‐old female C57BL/6J mice were obtained from the Guangdong Medical Laboratory Animal Center. The animals were housed in a specific pathogen‐free (SPF) facility under controlled environmental conditions (22°C, 50%–60% relative humidity, and a 12‐h light/dark cycle), with unrestricted access to food and water. A one‐week acclimatization period was allowed prior to initiating experimental procedures. All animal experiments were conducted in strict compliance with the ethical guidelines approved by the Ethics Committee of Guangzhou University of Chinese Medicine (No. 20240123007).

Mice were randomly distributed into 6 groups (*n* = 6): the sham‐operated group (CON), ovariectomized model group (OVX), positive control group (OVX + 0.1 mg/kg/day estradiol, E2), low‐dose ECG group (OVX + 25 mg/kg/day), middle‐dose ECG group (OVX + 50 mg/kg/day), and high‐dose ECG group (OVX + 100 mg/kg/day). For the OVX groups, bilateral ovariectomy was performed as previously described (Luo et al. 2020); the CON group underwent a sham surgical procedure without ovarian removal. After a two‐week recovery period, vaginal crystal violet staining was performed to verify the success of the OVX model (Figure S1). Subsequently, the CON group (CON + Saline) and OVX group (OVX + Saline) were orally gavaged (i.g.) with saline as vehicle control. The E2 group and ECG groups were orally gavaged (i.g.) with 0.1 mg/kg/day E2, 25 mg/kg/day ECG (low dose), 50 mg/kg/day ECG (middle dose), and 100 mg/kg/day ECG (high dose), respectively, for 12 consecutive weeks. At the end of the treatment period, serum and liver tissue samples were collected; the samples were either stored at −80°C or fixed in 4% paraformaldehyde for subsequent experimental analysis.

### 2.4 Cells Culture and Cell Counting Kit‐8 (CCK8) Assay

HepG2 cells were cultured in Dulbecco's Modified Eagle Medium (DMEM) supplemented with 10% fetal bovine serum (FBS) and were maintained at 37°C in a humidified incubator containing 5% CO_2_. The cells were seeded into 96‐well plates and exposed to varying ECG concentrations for 24 h. Following this, a premixed solution containing 10% CCK‐8 reagent (Glpbio Co, USA) was added to each well. Cell viability was evaluated by measuring the optical density (OD) at 450 nm with a microplate reader. The cellular metabolic disorder model was established in accordance with protocols described in previous studies (Cheng et al. 2020). Briefly, cells were seeded into culture plates and allowed to adhere overnight, followed by a 1‐h pretreatment with ECG at concentrations of 50, 100, and 200 μg/mL. After pretreatment, cells were exposed to a mixture of 50 μM PA and 200 μM OA for 24 h. Following the treatment period, both cells and culture medium were collected for subsequent analysis. All experiments were repeated at least three times.

### 2.5 Oil Red O (ORO) Staining in Cells

As described in the preceding Cell Culture section, treated cells were fixed in 10% formaldehyde for 1 h, rinsed with 60% isopropanol, and subsequently stained with freshly prepared ORO solution (Sigma, USA). After staining, excess dye was gently washed away with distilled water, and images were captured using a light microscope (Nikon, Japan) for visual assessment of lipid accumulation.

### 2.6 ROS Analysis

Intracellular ROS levels in HepG2 cells were measured using the ROS Detection Kit (Nanjing Jiancheng Bioengineering Institute, CHN). Fluorescence images were captured and analyzed using a Nikon fluorescence microscope (Nikon, USA).

### 2.7 Serum Indexes and Liver Lipids

Levels of triglycerides (TG), total cholesterol (TC), aspartate transaminase (AST), and alanine aminotransferase (ALT) in both serum samples and cell culture medium were measured using enzymatic assay kits (Nanjing Jiancheng Bioengineering Institute, CHN), following the manufacturer's instructions.

### 2.8 Histological Analysis

For histological analysis, liver tissues were fixed in 4% paraformaldehyde, embedded in paraffin, and sectioned into 5 μm slices for hematoxylin and eosin (H&E) staining to evaluate liver damage. For ORO staining, tissues were embedded in optimal cutting temperature (OCT) compound, cut into 5 μm sections, and stained with ORO solution to assess lipid accumulation. Images were captured using a digital pathology slide scanner (Konfoong Bioinformation Tech Co. Ltd, CHN).

### 2.9 Western Blotting

Total cellular and nuclear proteins were extracted separately using a commercial kit (Beyotime Co., Shanghai, CHN), following the manufacturer's instructions. Protein concentrations were determined using a bicinchoninic acid (BCA) assay kit (Beyotime Co., Shanghai, CHN) to ensure equal loading. Equal protein amounts (20–60 μg per lane) were loaded onto 10% sodium dodecyl sulfate‐polyacrylamide gel electrophoresis (SDS‐PAGE) gels, separated by electrophoresis, and then transferred onto polyvinylidene difluoride (PVDF) membranes. The PVDF membranes were blocked with 5% non‐fat milk for 1 h at room temperature, followed by overnight incubation at 4°C with primary antibodies. After washing with TBST, the membranes were incubated with secondary antibodies. Protein bands were visualized using an enhanced chemiluminescence (ECL) reagent (Guangdong Prochem Biotechnology Co. Ltd., China), and images were captured using a Molecular Imager (BIO‐RAD, USA).

### 2.10 Quantitative PCR (qPCR)

Total RNA was extracted from liver tissues or HepG2 cells using TRIzol reagent (Invitrogen, USA). cDNA was synthesized using a high‐capacity reverse transcription kit (Applied Biological Materials Inc., CAN). qPCR was performed using PowerUp SYBR Green Master Mix (Abclonal Co., CHN), and β‐actin was used as the internal reference gene to normalize gene expression levels. The sequences of the specific primers used in the assay are provided in Table S1.

### 2.11 Network Pharmacology Analysis

Potential targets of the active components of ECG in the drug were identified using the Traditional Chinese Medicine Systems Pharmacology Database and Analysis Platform (TCMSP, http://tcmspw.com/) following a previously established method (Huang et al. 2024). To ensure reliability, targets were selected based on an oral bioavailability (OB) of ≥ 30% and a drug‐likeness (DL) score of ≥ 0.18. MS‐related targets for *Homo sapiens* were retrieved from the TCMSP database, Online Mendelian Inheritance in Man (OMIM, https://omim.org/), and Genecards (https://www.genecards.org/). These targets were cross‐validated using UniProtKB IDs, and the corresponding protein names were extracted from the UniProt database (https://www.uniprot.org/). Finally, overlapping targets between the MS‐related proteins and the active components of ECG were identified, and these overlapping targets were designated as hub genes and analyzed using the online STRING database (https://string‐db.org/) to construct a protein–protein interaction (PPI) network based on predefined interaction score thresholds. The PPI network was visualized and analyzed using Cytoscape software (version 3.10.1). Functional enrichment analysis was performed using the Metascape database (http://metascape.org/), encompassing Gene Ontology (GO) terms (biological processes, molecular functions, and cellular components) and Kyoto Encyclopedia of Genes and Genomes (KEGG) pathways.

### 2.12 Molecular Docking Process

Molecular docking was performed following established methods (Li et al. 2024). The binding interactions between predicted active compounds and MS‐related targets were evaluated through docking simulations. Structural data (2D and 3D) for ECG's active components were retrieved from the PubChem database (https://pubchem.ncbi.nlm.nih.gov/). Docking analyses were carried out using AutoDockTools (version 1.5.7), and the results were visualized using Python 2.7.

### 2.13 Molecular Dynamics (MD) Simulations

MD simulations were further employed to investigate the stability of ECG's active components at key binding sites within the MS binding pocket (Huang et al. 2024; Li et al. 2024). All MD simulations were carried out using Gromacs 2020, which included four key stages: energy minimization, system heating, equilibration, and production MD. A 100 ns production run was executed, with trajectory data recorded at 10 ps intervals. Post‐simulation analysis was performed using the trjconv module. The binding free energy of the ligand–protein complex was calculated using the gmx_MMPBSA tool integrated into Gromacs 2020.

### 2.14 Statistical Analysis

Data analysis was conducted using GraphPad Prism (Version 9.5.0), with results expressed as means ± SEM. Statistical significance was assessed using one‐way ANOVA, and a *p*‐value of less than 0.05 was considered statistically significant.

## 3 Results

### 3.1 Analysis of ECG Extracts

**FIGURE 1:**
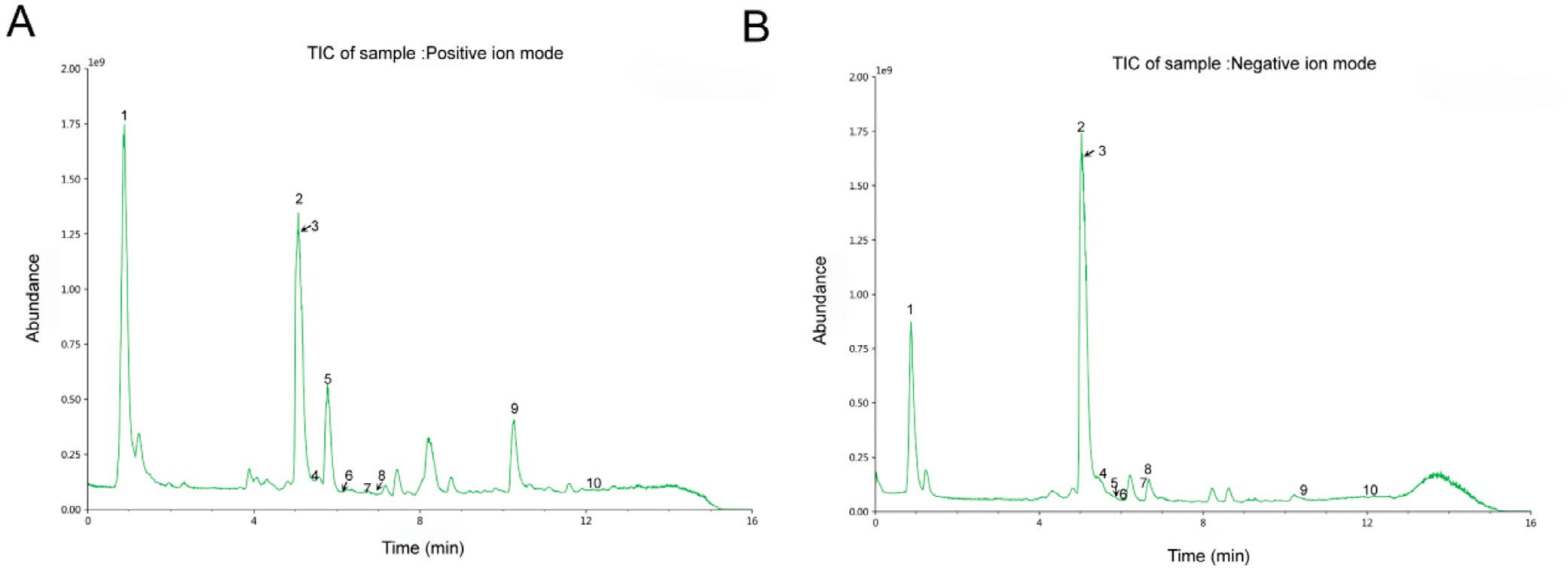
The UHPLC‐Q‐Exactive analytical platform revealed the characterization of 10 bioactive constituents in the ECG sample. (A) TIC of ECG sample: Positive ion mode. (B) TIC of ECG sample: Negative ion mode. Compounds were identified as Stachydrine (1), Naringin (2), Narirutin (3), Dihydrokaempferol (4), Dihydrodaidzein (5), Bergaptol (6), Naringenin chalcone (7), Apigenin (8), Imperatorin (9), Auraptene (10).

Key constituents in ECG samples were identified using UHPLC‐Q‐Exactive analysis. By comprehensively evaluating retention time, mass error (ppm), fragment ions, observed (m/z), and ion mode information, and the mass spectrometry database as well as the TCM Integrated Database, 10 bioactive components were successfully characterized (Figure 1, Table S2). These components include Stachydrine, Naringin, Narirutin, Dihydrokaempferol, Dihydrodaidzein, Bergaptol, Naringenin chalcone, Apigenin, Imperatorin, and Auraptene. This systematic chemical profiling provides critical chemical insights for investigating ECG's therapeutic potential in addressing metabolic disorders.

### 3.2 ECG Alleviated Lipid Deposition in OA&PA‐Induced HepG2 Cells

**FIGURE 2:**
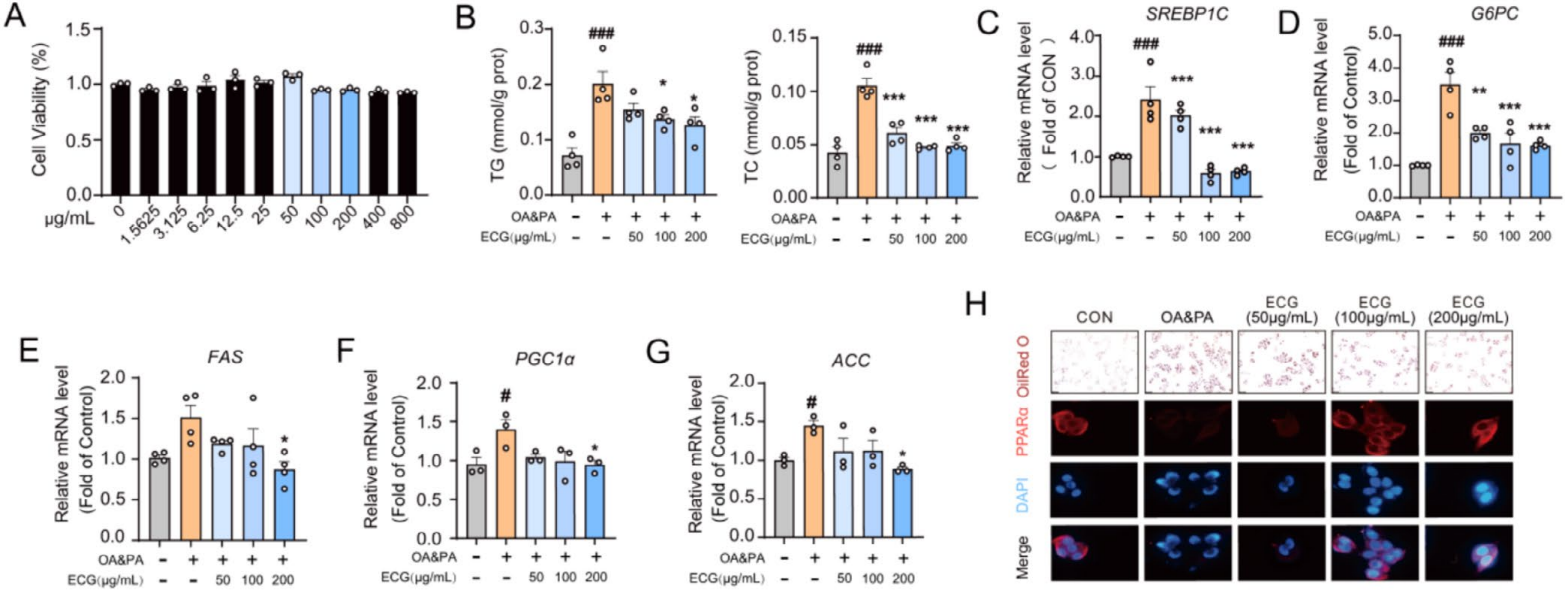
ECG alleviated lipid deposition in OA&PA‐induced HepG2 cells. (A) The cytotoxicity of ECG on HepG2 cells. (B) TG and TC levels in HepG2 cells. (D‐G) Relative mRNA expression levels of *SREBP1C, G6PC, FAS, PGC1α*, and *ACC*. (H) Representative images of Oil Red O staining (20×) and representative immunofluorescence images of PPARα‐positive in HepG2 cells (100×). Data are presented as means ± SEM (*n* = 3–4). Compared to the OA&PA group, *p < 0.05, **p < 0.01, and ***p < 0.001. Compared to the control group, ^#^ *p* < 0.05, and ^###^ *p* < 0.001.

In vitro experiments were conducted using OA&PA‐induced HepG2 cells to investigate the role of ECG in improving cellular lipid deposition. As shown in Figure 2A, treating HepG2 cells with ECG at concentrations up to 800 μg/mL for 24 h had no significant effect on cell viability. Results indicated that ECG treatment (50, 100, and 200 μM) significantly reversed the elevation in TG and TC levels in OA&PA‐induced HepG2 cells in a dose‐dependent manner, suggesting that ECG exerts a beneficial effect on lipid deposition (Figure 2B). Additionally, compared to the control group, down‐regulated mRNA levels of lipogenesis‐ and gluconeogenesis‐related genes such as SREBP1C, G6PC, FAS, PGC1α, and ACC were observed in the ECG‐treated groups (Figure 2C–G). Moreover, ECG improved OA&PA‐induced lipid accumulation, which was confirmed by ORO staining (Figure 2H). Immunofluorescence results further showed that compared with the control group, the expression of PPARα was significantly decreased in the OA&PA‐induced group, while ECG treatment markedly up‐regulated PPARα expression (Figure 2H).

### 3.3 ECG Alleviated Inflammation in OA&PA‐Induced HepG2 Cells

**FIGURE 3:**
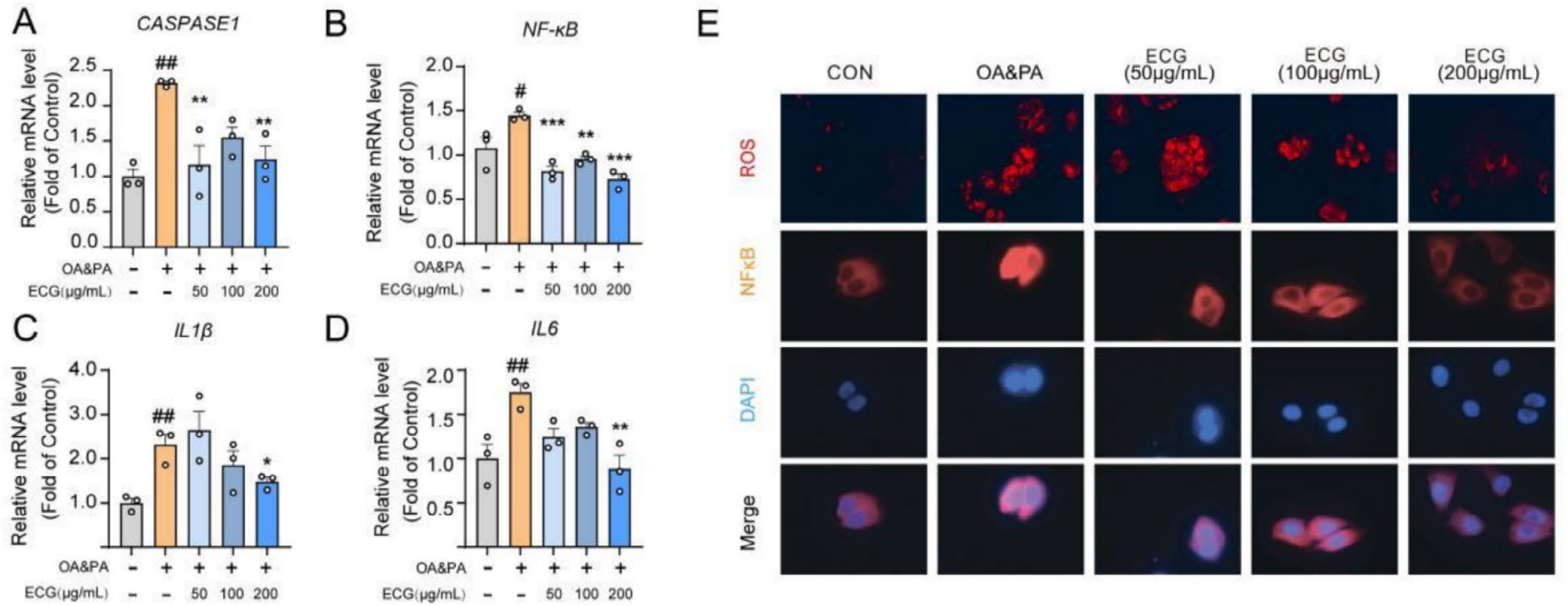
ECG alleviated inflammation in OA&PA‐induced HepG2 cells. (A–D) Relative mRNA expression levels of hepatic genes related to inflammation, including CASPASE1, NF‐κB, IL1β, and IL6. (E) Representative images of ROS‐stained fluorescence images for detecting ROS levels and representative immunofluorescence images of NF‐κB‐positive in HepG2 cells (100×). Data are presented as means ± SEM (*n* = 3). Compared to the OA&PA group, *p < 0.05, **p < 0.01, and ***p < 0.001. Compared to the control group, ^#^ *p* < 0.05, and ^##^ *p* < 0.01.

To investigate the effect of ECG on inflammation in OA and PA‐induced HepG2 cells, further experiments were conducted. During OA and PA‐induced inflammatory stimulation, ECG effectively inhibited the expression of inflammatory genes, particularly *Caspase1, NF‐κB, IL1β*, and *IL6* (Figure 3A–D). Notably, immunofluorescence analysis yielded similar results, showing that ECG significantly inhibited the nuclear translocation of NF‐κB and reduced intracellular ROS production (Figure 3E).

### 3.4 ECG Alleviated NLRP3 Inflammasome Activation in OA&PA‐Induced HepG2 Cells

**FIGURE 4:**
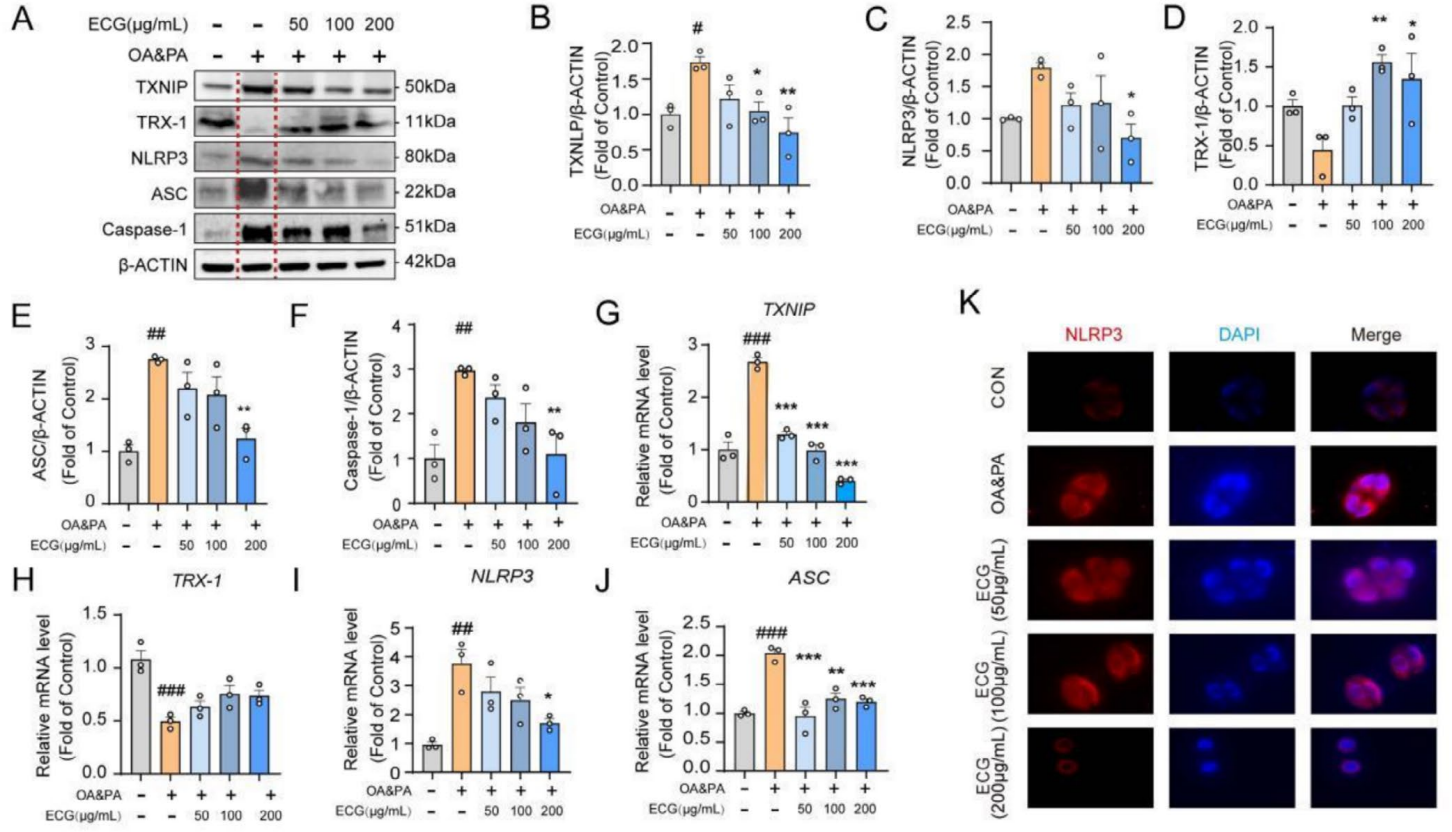
ECG suppressed NLRP3 inflammasome in OA and PA‐induced HepG2 cells. (A–F) Representative immunoblotting and quantification of TXNLP, TRX‐1, NLRP3, ASC, and Caspase‐1 proteins in HepG2 cells. (G–J) Relative mRNA expression levels of hepatic genes related to NLRP3 inflammasome, including *TXNIP, TRX‐1, NLRP3*, and *ASC*. (K) Representative immunofluorescence images of NLRP3‐positive in HepG2 cells (100×). Data are presented as means ± SEM (*n* = 3). Compared to the OA and PA group, *p < 0.05, **p < 0.01, and ***p < 0.001. Compared to the control group, ^#^ *p* < 0.05, ^##^ *p* < 0.01, and ^###^ *p* < 0.001.

To investigate the effect of ECG on the NLRP3 inflammasome, experiments such as immunofluorescence, western blot analysis, and qPCR were conducted. As shown in Figure 4A–F, OA&PA up‐regulated the expression levels of TXNIP, NLRP3, ASC, and Caspase‐1 proteins, while ECG significantly reduced the expression level of those proteins. During OA&PA intervention, ECG effectively inhibited the expression of NLRP3 inflammasome‐related genes, including *TXNIP, TRX‐1, NLRP3*, and *ASC* (Figure 4G–J). Consistent with these results, immunofluorescence analysis also showed that ECG significantly down‐regulated the protein level of NLRP3 (Figure 4K).

### 3.5 ECG Mitigated Hepatic Metabolic Disorder in OVX Mice

**FIGURE 5:**
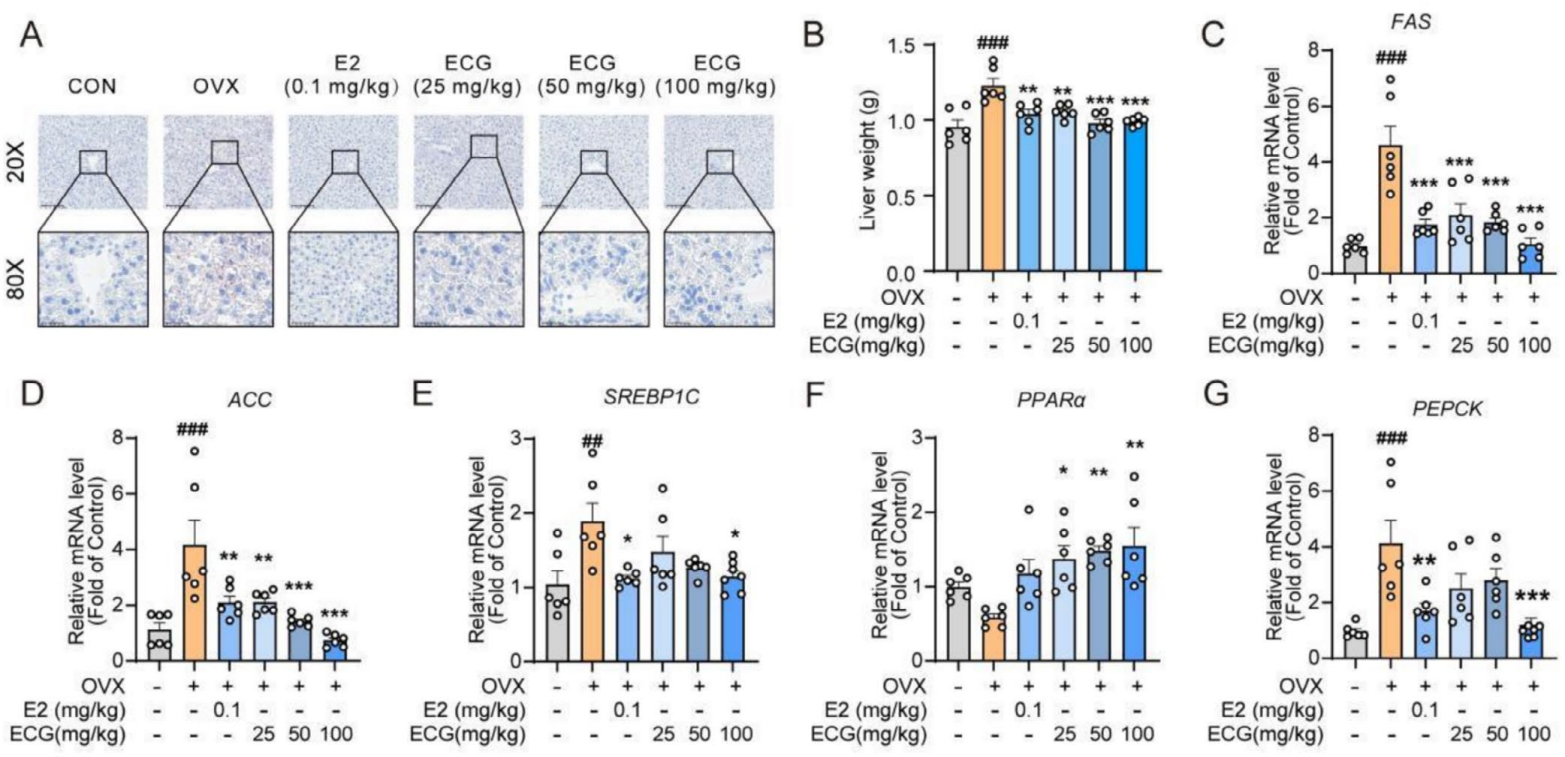
ECG mitigated lipid deposition in the liver in OVX mice. (A) Representative images of oil red O staining in liver tissues (20×, 80×). (B) Liver weight. (C–G) Relative mRNA expression levels of *FAS, ACC, SREBP1C, PPARα* and *PEPCK* (*n* = 6). Data are presented as means ± SEM (*n* = 6). Compared to the OVX group, *p < 0.05, **p < 0.01, and ***p < 0.001. Compared to the CON group, ^##^ *p* < 0.01 and ^###^ *p* < 0.001.

After initially identifying the role of ECG in restoring lipid metabolism homeostasis in vitro, we further explored its therapeutic effect in vivo. ORO staining revealed excessive lipid accumulation in the livers of the OVX group, whereas lipid accumulation was reduced in the livers of the E2 and ECG treatment groups (Figure 5A). The OVX group exhibited increased liver weight, and this increase was reduced by treatment with ECG or E2 (Figure 5B). Additionally, ECG at the 100 mg/kg dose significantly suppressed the expression of genes involved in lipogenesis, including *FAS, ACC*, and *SREBP1C* (Figure 5C–E), as well as *Pepck* (a gene involved in gluconeogenesis) (Figure 5F). Furthermore, compared with the OVX group, ECG treatment up‐regulated the expression of PPARα (a gene related to fatty acid oxidation) (Figure 5G).

### 3.6 ECG Suppressed Hepatic Inflammation in OVX Mice

**FIGURE 6:**
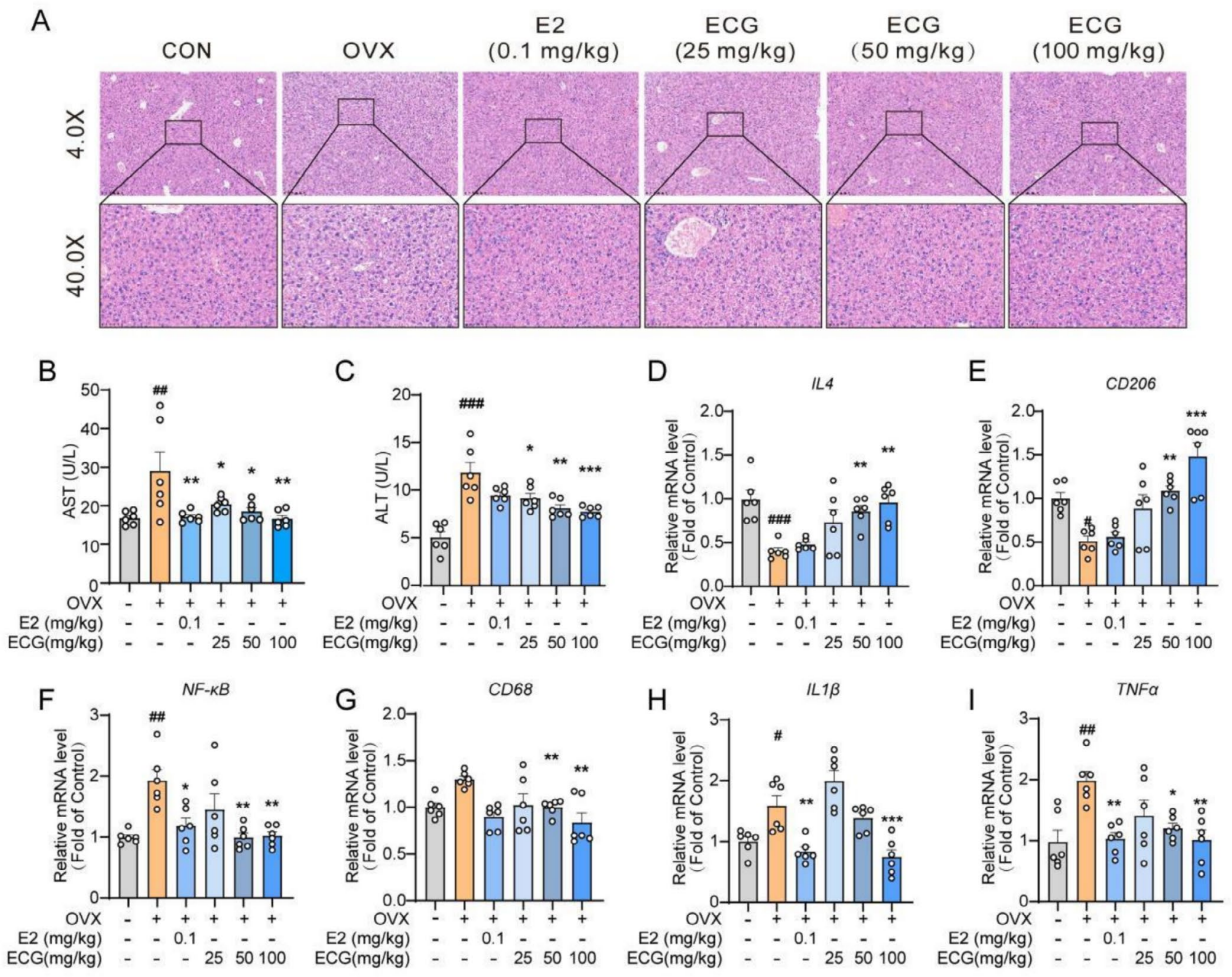
ECG mitigated inflammation in the liver in OVX mice. (A) Representative images of H&E staining in liver tissues (4.0×, 40.0×). (B, C) Serum ALT and AST levels. (D–I) Relative mRNA expression levels of IL4, CD206, NF‐κB, CD68, IL1β, and TNFα. Data are presented as means ± SEM (*n* = 6). Compared to the OVX group, *p < 0.05, **p < 0.01, ***p < 0.001 compared with the OVX group; ^#^ *p* < 0.05, ^##^ *p* < 0.01, ^###^ *p* < 0.001 compared with the CON group.

H&E staining of liver tissue showed that hepatocyte vacuoles in the OVX group were larger than those in the CON group, which indicated increased hepatic steatosis following OVX surgery (Figure 5A). After 12 weeks of treatment with ECG and E2, the hepatocyte vacuoles caused by lipid accumulation were reduced. All therapeutic groups, including those treated with E2 (0.1 mg/kg) and ECG (25, 50, 100 mg/kg), exhibited decreased vacuolization (Figure 6A). Serum ALT and AST levels in the OVX group were higher than those in the CON group, which indicated that estrogen deficiency exacerbated liver damage, while ECG intervention significantly reduced serum ALT and AST levels (Figure 6B,C). Compared with the CON group, the OVX group showed increased mRNA expression levels of *IL1β, TNFα, NF‐κB*, and *CD68* in the liver, while the expression levels of *IL4* and *CD206* were decreased, indicating an enhanced inflammatory response in the liver (Figure 6D–I). As anticipated, following ECG intervention, the mRNA expression levels of *IL1β, TNFα, NF‐κB*, and *CD68* were down‐regulated, and mRNA expression levels of *IL4* and *CD206* were up‐regulated (Figure 6D–I).

### 3.7 ECG Suppressed Hepatic NLRP3 Inflammasome Activation in OVX Mice

**FIGURE 7:**
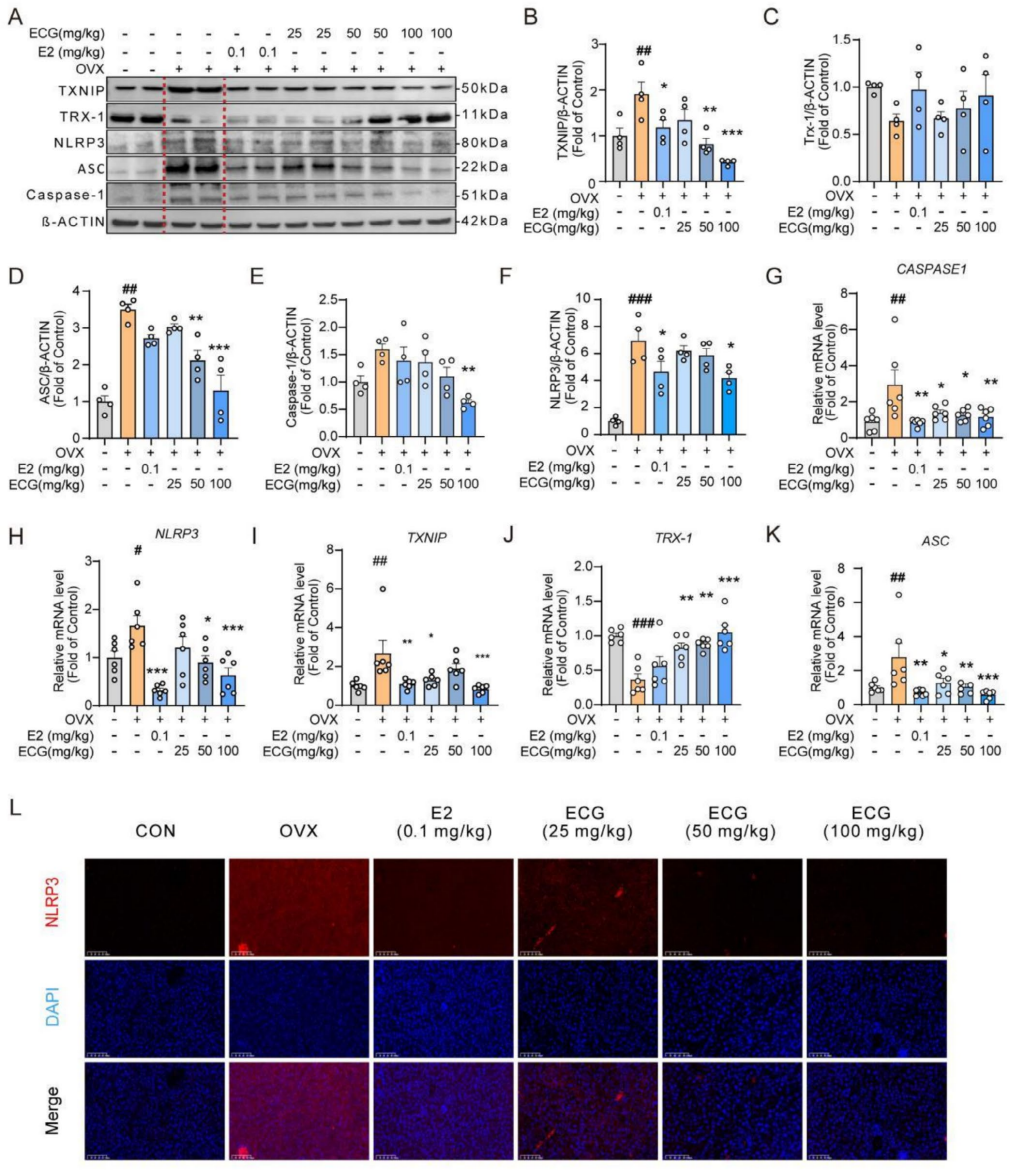
ECG suppressed NLRP3 inflammasome in the liver in OVX mice. (A–F) Representative immunoblotting and quantification of TXNIP, TRX‐1, ASC, Caspase‐1, and NLRP3. (H–K) Relative mRNA expression levels of *NLRP3, TXNIP, TRX‐1*, and *ASC*. (L) Representative immunofluorescence images of NLRP3‐positive in the liver tissues (20.0×). Data are presented as means ± SEM (*n* = 6). Compared to the OVX group, *p < 0.05, **p < 0.01, ***p < 0.001 compared with the OVX group; ^#^ *p* < 0.05, ^##^ *p* < 0.01, ^###^ *p* < 0.001 compared with the CON group.

As shown in Figure 7A–F, the protein levels of TXNIP, NLRP3, ASC, and Caspase‐1 in the OVX group were higher than those in the CON group, while ECG treatment down‐regulated these proteins. qPCR results also showed a consistent trend that OVX increased the mRNA levels of *TXNIP, NLRP3, ASC*, and *CASPASE1*, and ECG treatment reduced these elevated mRNA levels (Figure 7G–K). Compared to the CON group, the OVX group exhibited down‐regulated protein and mRNA levels of TRX‐1, while ECG treatment up‐regulated TRX1 at both the protein and mRNA levels (Figure 7A,C,J). Furthermore, immunofluorescence analysis showed that NLRP3 expression was up‐regulated in the OVX group compared with the CON group, and ECG treatment reduced this elevated NLRP3 expression (Figure 7L).

### 3.8 ECG Regulates PI3K/AKT Signaling

**FIGURE 8:**
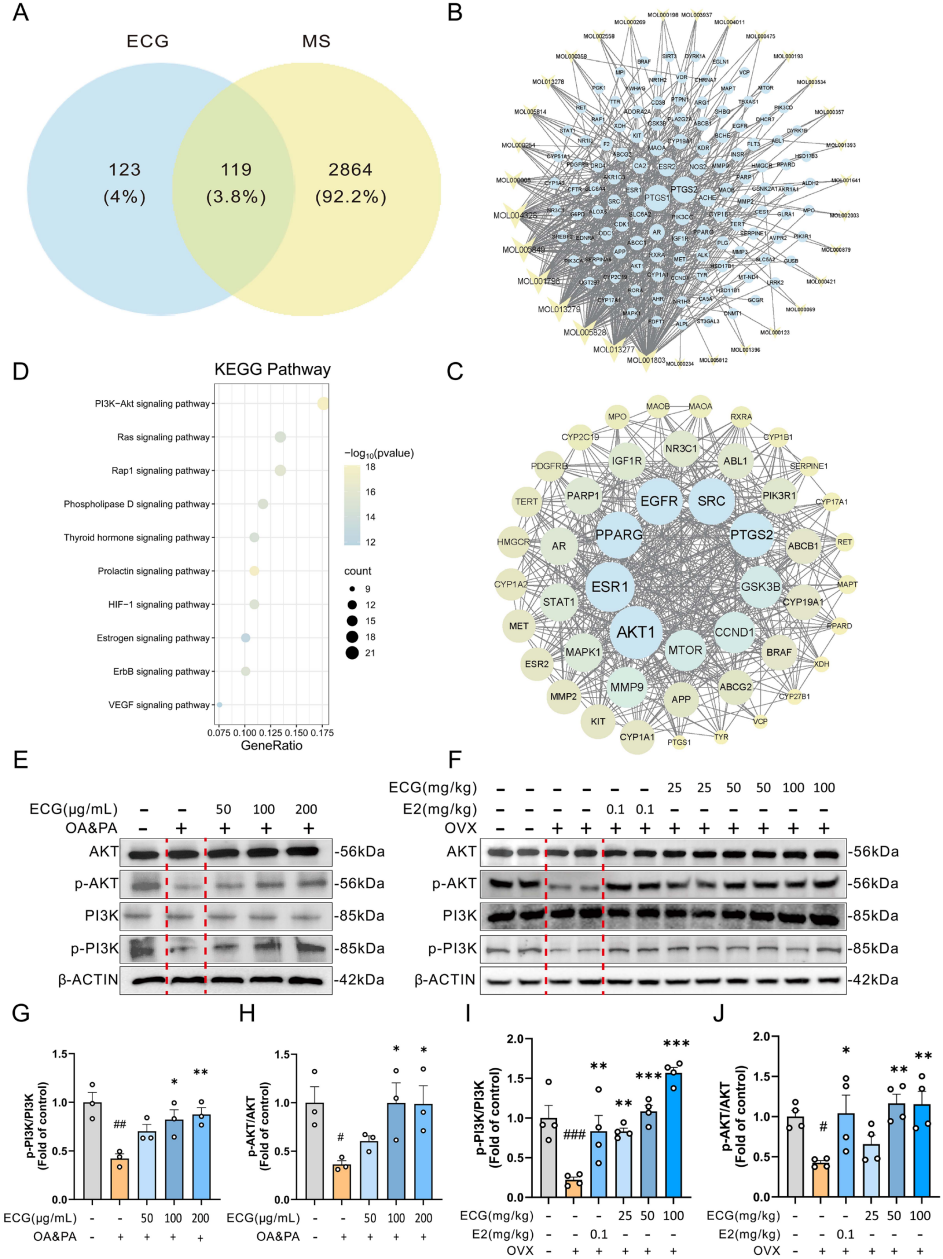
ECG regulates PI3K/AKT signaling. (A) Venn diagram. (B) The ECG‐active components‐related targets network. (C) KEGG pathway enrichment analysis of core targets. (D) The PPI network of ECG and MS core targets, and edges represent protein–protein association, and nodes represent proteins (the colors from dark to bright represent the degree of binding between proteins). (E, F) Representative immunoblotting and quantification of AKT, p‐AKT, PI3K, and p‐PI3K in HepG2 cells and OVX mice. (G, H) Western blot quantitative analysis of AKT, p‐AKT, PI3K, and p‐PI3K protein expression in the OA&PA‐induced HepG2 cell model. (I, J) Western blot analysis of AKT, p‐AKT, PI3K, and p‐PI3K protein expression in liver tissues of OVX mice, with densitometric quantification of band intensities. Data are presented as mean ± SEM (HepG2, *n* = 3; OVX mice, *n* = 4). For HepG2 cells: *p < 0.05, **p < 0.01, ***p < 0.001 vs. OA&PA group; ^#^ *p* < 0.05, ^##^ *p* < 0.01, ^###^ *p* < 0.001 vs. control group. For OVX mice: *p < 0.05, **p < 0.01, ***p < 0.001 vs. OVX group; ^#^ *p* < 0.05, ^##^ *p* < 0.01, ^###^ *p* < 0.001 vs. CON group.

The Swiss Target Prediction database was used to predict targets, leading to the identification of 123 ECG‐related targets. Venn diagram analysis of the intersection between ECG‐ and MS‐related targets showed that among the relevant targets, 119 were shared by both ECG and MS, indicating that they may potentially serve as therapeutic targets for ECG (Figure 8A). As shown in Figure 8B, 31 active components of ECG were identified, 119 potential targets were collected, and a network of targets related to ECG active components was constructed. To explore the relationship between biological functions and target proteins, the top 10 KEGG pathways with the highest significance (based on log*p* values) were analyzed for these 119 shared genes, providing insights into the potential mechanisms through which ECG alleviates MS. The PI3K/AKT signaling pathway, which plays an important role in inflammation and glucose‐lipid metabolism, ranked first, indicating it may be the key mechanism underlying ECG's therapeutic effects on MS (Figure 8C). The PPI network diagram was analyzed using the CytoNCA plugin in Cytoscape 3.9.1 to assess node connectivity, betweenness centrality, and closeness centrality. Ultimately, the top 10 key targets were identified, which included AKT1, ESR1, PPARG, EGFR, SRC, PTGS2, GSK3β, CPT1A, CCDN1, and MTOR (Figure 8D). Western blot analysis showed that ECG intervention significantly restored the impaired phosphorylation of PI3K and AKT in OA&PA‐induced HepG2 cells in a dose‐dependent manner (Figure 8E). Consistently, in vivo results revealed that liver tissues from OVX mice exhibited markedly reduced phosphorylation of PI3K and AKT compared with those from the CON group, whereas ECG administration effectively reversed these alterations in a dose‐dependent manner. Quantitative densitometric analysis further confirmed that ECG treatment significantly modulated the expression levels of AKT, p‐AKT, PI3K, and p‐PI3K, with clear statistical differences compared with the OVX groups (Figure 8F–J).

### 3.9 Interactions and Dynamics Stability Simulation Between Active Ingredients With Targets

**FIGURE 9:**
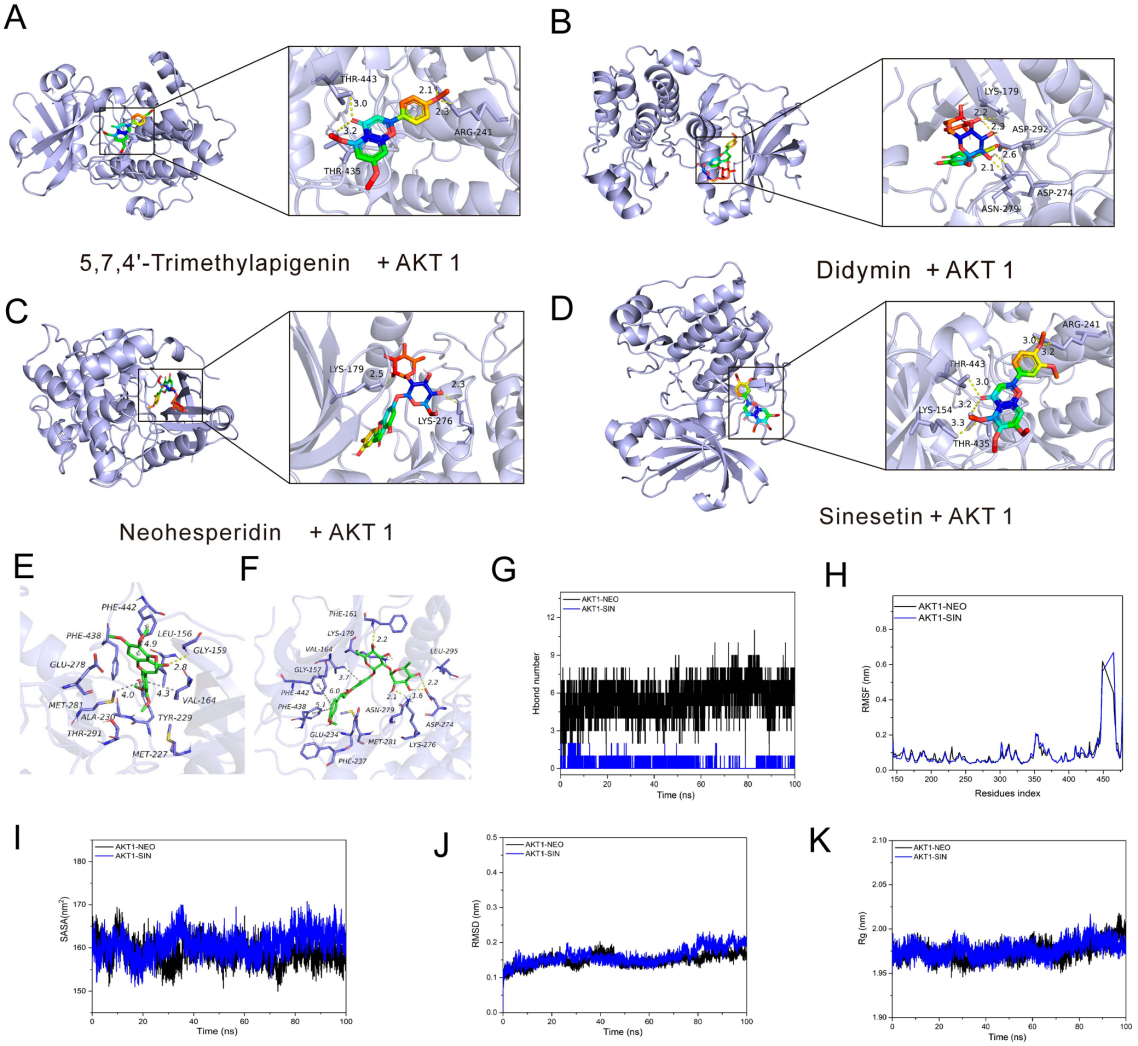
Interactions and dynamics stability simulation between active ingredients with targets. (A–D) Docking model of the key components of ECG with AKT1, including 5,7,4′‐Trimethylapigenin, Didymin, Neohesperidin (NEO), and Sinesetin (SIN). (E, F) Images of AKT1‐NEO and AKT1‐SIN complexes after MD simulation. (G–H) MD simulation analysis showing hydrogen bond number, RMSF, SASA, RMSD, and Rg of SIN and NEO with AKT1.

Network pharmacology analysis suggested that key components of ECG, such as Sinensetin (SIN), Isosinensetin, Neohesperidin (NEO), 5,7,4′‐Trimethylapigenin, and Didymin, may play crucial roles in the treatment of MS in this study. To further explore the mechanism of ECG in MS, molecular docking was performed. The binding of these components with AKT1, which is a key target involved in inflammatory signaling and metabolism, showed total scores below −7 kcal/mol and C‐scores close to 5, indicating strong activity between them (Figure 9A–D). Results also demonstrated that most compounds exhibited favorable binding energy, with intermolecular forces including conventional hydrogen bonds, alkyl interactions, and π‐alkyl interactions (Figure 9A–D). Given the strong binding activity between ECG's active components and AKT1 revealed by molecular docking, MD simulations were conducted. NEO formed hydrogen bonds with key residues of the AKT1 protein binding site, such as PHE‐161, LYS‐276, and ASP‐274, and also engaged in hydrophobic interactions with PHE‐161, VAL‐164, PHE‐442, PHE‐438, PHE‐237, MET‐281, and LEU‐295 (Figure 9E). Meanwhile, SIN formed hydrogen bonds with GLY‐159 of AKT1 and established hydrophobic interactions with MET‐227, MET‐229, MET‐281, PHE‐438, PHE‐442, LEU‐156, VAL‐164, and TYR‐229 (Figure 9F). NEO, which contains multiple hydrogen bond donor/acceptor groups, formed 3–5 hydrogen bond interactions with amino acid residues in the AKT1 binding pocket. In contrast, SIN is a more hydrophobic compound with only hydrogen bond acceptors, and it maintained at least 1 hydrogen bond with AKT1 (Figure 9G). Nevertheless, these persistent hydrogen bonds played a crucial role in stabilizing the small molecules. As shown in Figure 9H, the Root Mean Square Deviation (RMSD) plot indicates that the average RMSD of the AKT1‐SIN and AKT1‐NEO complexes was less than 2 Å, with the AKT1‐NEO complex displaying slightly better stability than the AKT1‐SIN complex. According to the data, the solvent‐accessible surface area (SASA) of the AKT1‐NEO complex showed a slight decrease, while the SASA of the AKT1‐SIN complex showed a slight increase, but the overall change is not significant (Figure 9I–K). The binding free energies of SIN and NEO with the AKT1 protein were −14.592 ± 3.016 kcal/mol and −19.166 ± 4.469 kcal/mol, respectively, with van der Waals forces playing a dominant role (Table S3), indicating that the compounds can stably bind within the protein's binding pocket and exhibit strong van der Waals interactions with surrounding residues.

## 4 Discussion

Although HRT for postmenopausal women can reduce the risk of the prevalence of metabolic syndrome, the benefits and risks of HRT remain controversial due to the possible risks of cardiovascular disease, breast cancer, venous thromboembolism, stroke, dementia, and other chronic diseases (Cho et al. 2023; Lobo 2017). Traditional Chinese medicine exhibits unique advantages in treating MS due to its broad multi‐component and multi‐target characteristics. As a traditional Chinese medicinal material, ECG is not only a valuable therapeutic agent but also a common daily food, reflecting the core concept of “medicine and food sharing the same origin” in traditional Chinese medicine (Xu et al. 2024). The current study is the first to demonstrate that ECG can improve metabolic disorder by inhibiting inflammatory responses and NLRP3 inflammasome activation as well as modulating the PI3K/AKT1 signaling pathway. The pharmacological effects of ECG have been verified via in vitro and in vivo experiments, providing new insights into the potential application of ECG in managing menopausal metabolic syndrome.

Aging‐induced hepatic alteration including diminished perfusion, volumetric regression and impaired regenerative capacity, create a permissive microenvironment for lipidomic dysregulation, wherein even ostensibly benign steatosis progresses via mitochondrial‐peroxisomal oxidative derangements that generate pathogenic ROS and inflammatory cascades (Brady 2015). As a critical regulator of systemic metabolism, liver functionality is markedly compromised under estrogen‐deficient conditions (Xue et al. 2024), which increases the risk of MAFLD and may further progress to metabolic dysfunction‐associated steatohepatitis and liver fibrosis. The OVX model is commonly used to study metabolic diseases associated with estrogen deficiency, and it shows fundamental changes in insulin sensitivity, glucose tolerance, and body weight (Araujo et al. 2023; Baudin et al. 2024). Serum AST and ALT levels are often used as indicators to evaluate liver function and liver injury (Xie et al. 2019). In the current study, OVX mice exhibited abnormalities in liver function, hepatic fat deposition, and lipid metabolism, which further demonstrates the association between estrogen deficiency and hepatic metabolic disorders. Over the past few decades, data from clinical and experimental studies have shown that E2 not only plays a significant role in sexual development and reproduction but also greatly contributes significantly to maintaining blood glucose and lipid homeostasis (Gravholt et al. 1998; Liu et al. 2013). In the current study, E2 was used as a positive control, and results showed that ECG exerts a similar therapeutic effect to E2 in improving hepatic inflammation and lipid accumulation in OVX mice.

To further investigate the role of ECG in MS, this study employed an in vitro cell model using OA&PA induced HepG2 cells, which has been widely used in studies of lipid metabolism, cholesterol synthesis, and lipid accumulation, making it a classic model for studying liver metabolic disorders and lipid deposition in our previous studies and those of other researchers (Wang et al. 2022; Zhang et al. 2024). In the current study, we found that ECG significantly reduced lipid accumulation and the expression of inflammation marker expression in OA&PA‐induced HepG2 cells, which was consistent with the results of in vivo experiment using OVX mice, indicating that ECG exhibits good therapeutic effects in both in vivo and in vitro models of MS.

In both obese murine models and human liver tissues, enhanced expression of NLRP3 inflammasome components, increased CASPASE‐1 activity, and elevated levels of IL‐1β have been observed, correlating directly with the severity of metabolic syndrome phenotypes (Ives et al. 2015). The NLRP3 inflammasome as a sensor for metabolic danger signals that accumulate during obesity, thereby inducing the production of various cytokines and chemokines. Previous studies have highlighted the significant involvement of the PI3K/AKT1 signaling pathway in inflammatory responses and its upstream regulatory role in NLRP3 inflammasome activation (Wang et al. 2019). It is well established that the PI3K/AKT1 signaling pathway regulates multiple celluar functions, including cell survival, proliferation, metabolism, and many other biological processes (Yu and Cui 2016). When cells are stimulated by inflammatory signals, AKT1 can phosphorylate IκB kinase, leading to the degradation of IκB protein and the release of NF‐κB into the nucleus, which then binds to the promoter regions of many inflammation‐related genes, such as IL1β, tumor TNFα, etc. (Cheng et al. 2023). Meanwhile, the PI3K/AKT1 pathway can also regulate the activity of PPARG, an important regulator of adipocyte differentiation and lipid metabolism, thereby influencing lipid metabolism processes (Li et al. 2020). Furthermore, NF‐κB activation can further enhance the expression of the NLRP3 inflammasome, thereby triggering pyroptosis (Li et al. 2019; Ma et al. 2018). In the current study, potential targets including PI3K, AKT1 were identified via network pharmacology analysis. Experiments including molecular docking, molecular dynamics simulation, and western blotting all verified the efficiency of ECG in regulating the PI3K/AKT1 signaling pathways.

Through network pharmacology analysis, the current study identified the potential active components of ECG, including sinensetin, isosinensetin, nobiletin, neohesperidin, 5,7,4′‐trimethylapigenin, and didymin, which were also detected in UHPLC‐Q‐exactive analysis in our previous study (Xu et al. 2024). However, although our study demonstrated stable and strong binding of sinensetin and neohesperidin to the potential targets AKT1, the specific effects of these two components on MS have not been reported. Previous studies have found that sinensetin and neohesperidin can affect the lipid metabolism in adipocytes or mice (Kang et al. 2013; Wang et al. 2020). In summary, ECG itself and its derived components sinensetin and neohesperidin are expected to be developed as functional foods or therapeutic agents for the prevention of MS, and this direction will be the focus of our future research.

## 5 Conclusion

In conclusion, this study confirmed that ECG ameliorates MS by inhibiting inflammatory responses, suppressing NLRP3 inflammasome activation, and modulating the PI3K/AKT1 signaling. These findings emphasize the potential role of ECG in ameliorating MS as well as other metabolic diseases and provide evidence for the novel functional applications of ECG.

## Author Contributions

**Tingting Chen:** investigation (equal), visualization (equal), writing – review and editing (equal). **Xiaoqin Wu:** investigation (equal), writing – review and editing (equal). **Danna Wang:** investigation (equal), writing – review and editing (equal). **Tianqi Cui:** investigation (equal). **Jiayu Li:** investigation (equal). **Ziyi Zhang:** investigation (equal). **Min Qiu:** investigation (equal). **Chenlu Ma:** investigation (equal). **Hengrui Liu:** visualization (equal). **Yong Gao:** visualization (equal), writing – review and editing (equal). **Jiangtao Zeng:** investigation (equal), visualization (equal), writing – review and editing (equal). **Hang Li:** visualization (equal), writing – review and editing (equal). **Jiawen Huang:** conceptualization (equal), visualization (equal), writing – review and editing (equal).

## Disclosure

The authors have nothing to report.

## Conflicts of Interest

The authors declare no conflicts of interest.

## Supporting information


**Table S1:** Primers used in qPCR analysis.
**TABLE S2:** Compounds from ECG were identified by UHPLC‐Q‐Exactive analysis.
**TABLE S3:** The binding energy by MMGBSA. Binding free energy components (kcal/mol) calculated by MM/GBSA for the complexes of ATK1 with SIN and NEO.
**FIGURE S1:** ECG reversed the alterations in the estrous cycle induced by ovariectomy (OVX) in mice. Representative images of vaginal crystal violet staining in mice before sacrificed (scale bar = 100 px).

## Data Availability

Data available on request from the authors.
