# Supplementary material for: Exocarpium Citri Grandis Ameliorates Metabolic Disorders by Inhibiting Inflammatory Responses and NLRP3 Inflammasome Activation
Source: Food Sci Nutr. 2025 Oct 28;13(11):e71074. doi: 10.1002/fsn3.71074 (PMC12567642; doi:10.1002/fsn3.71074)

**Supplementary Table 1**. Primers used in qPCR analysis.

| **Gene** | **Forward Primer** | **Reward Primer** |
| --- | --- | --- |
| *IL6* | TAGTCCTTCCTACCCCAATTTCC | TTGGTCCTTAGCCACTCCTTC |
| *IL1β* | GCA ACTGTTCCTGAACTCAACT | ATCTTTTGGGGTCCGTCAACT |
| *NF-κB* | ATGGCAGACGATGATCCCTAC | TGTTGACAGTGGTATTTCTGGTG |
| *TNFα* | CCCTCACACTCAGATCATCTTCT | GCTACGACGTGGGCTACAG |
| *CASPASE1* | ACAAGGCACGGGACCTATG | TCCCAGTCAGTCCTGGAAATG |
| *SREBP1C* | TGACCCGGCTATTCCGTGA | CTGGGCTGAGCAATACAGTTC |
| *G6PC* | CGCCATGTTCATTGTGGTCAT | ATTCTCCTTCATCGCACAGTG |
| *FAS* | TATCAAGGAGGCCCATTTTGC | TGTTTCCACTTCTAAACCATGCT |
| *ACC* | ATGGGCGGAATGGTCTCTTTC | TGGGGACCTTGTCTTCATCAT |
| *PGC1α* | TATGGAGTGACATAGAGTGTGCT | CCACTTCAATCCACCCAGAAAG |
| *PPARα* | AGAGCCCCATCTGTCCTCTC | ACTGGTAGTCTGCAAAACCAAA |
| *PEPCK* | CTGCATAACGGTCTGGACTTC | CAGCAACTGCCCGTACTCC |
| *CD68* | TGTCTGATCTTGCTAGGACCG | GAGAGTAACGGCCTTTTTGTGA |
| *IL4* | GGTCTCAACCCCCAGCTAGT | GCCGATGATCTCTCTCAAGTGAT |
| *CD206* | CTCTGTTCAGCTATTGGACGC | CGGAATTTCTGGGATTCAGCTTC |
| *TXNIP* | GGCCGGACGGGTAATAGTG | AGCGCAAGTAGTCCAAAGTCT |
| *TRX-1* | CATGCCGACCTTCCAGTTTTA | TTTCCTTGTTAGCACCGGAGA |
| *NLRP3* | TGTGAGAAGCAGGTTCTACTCT | TGTAGCGACTGTTGAGGTCCA |
| *β-actin* | GGCTGTATTCCCCTCCATCG | CCAGTTGGTAACAATGCCATGT |

**Supplementary Table 2**. Compounds from ECG were identified by UHPLC-Q-Exactive analysis.

| **NO.** | **RT**  **(min)** | **Adducts** | **Formula** | **Mass Error**  **(ppm)** | **Identification** | **Fragment Ions** | **Observed**  **m/z** | **Ion mode** |
| --- | --- | --- | --- | --- | --- | --- | --- | --- |
| 1 | 1.112 | M+Na | C7H13NO2 | -1.2 | Stachydrine | 166.08424 | 166.08396 | Positive |
| 2 | 5.037 | 2M-H | C27H32O14 | 2.33 | Naringin | 271.06046, 459.11462, 579.1709, 580.17383 | 1159.34839 | Negative |
| 3 | 5.069 | M+H | C27H32O14 | -1.2 | Narirutin | 195.0292, 219.0293, 273.0762, 315.0872, 339.0872, 383.1131, 401.1245, 417.1193, 419.1349, 435.1295 | 581.18718 | Positive |
| 4 | 5.523 | M-H | C15H12O6 | 2.79 | Dihydrokaempferol | 125.02401, 151.00317, 243.06557, 259.06052, 287.05518 | 287.05526 | Negative |
| 5 | 5.887 | M+H | C15H12O4 | 0 | Dihydrodaidzein | 131.04967, 153.01857, 171.02919, 257.08136 | 257.08084 | Positive |
| 6 | 6.264 | M+H | C11H6O4 | -0.49 | Bergaptol | 147.04436, 203.03432 | 203.03404 | Positive |
| 7 | 6.728 | M+H | C15H12O5 | -0.37 | Naringenin chalcone | 147.0444, 153.01854, 273.07623 | 273.07579 | Positive |
| 8 | 9.016 | M+H | C18H16O5 | 7.98 | Apigenin 5,7,4'-trimethyl ether | 313.10507, 313.27573, 314.10806 | 313.10462 | Positive |
| 9 | 10.418 | M+H | C16H14O4 | 0 | Imperatorin | 203.03439 | 271.09655 | Positive |
| 10 | 12.14 | M+H | C19H22O3 | 0 | Auraptene | 81.07058, 163.03931, 184.8893 | 299.1642 | Positive |

**Supplementary Table 3**.The binding energy by MMGBSA. Binding free energy components (kcal/mol) calculated by MM/GBSA for the complexes of ATK1 with SIN and NEO.

| Type | ATK1-SIN | ATK1-NEO |
| --- | --- | --- |
| *E_VDW_* | -30.499±2.707 | -26.034±2.231 |
| *E_ELE_* | -35.939±2.973 | -14.834±2.706 |
| *E_GB_* | 51.116±5.561 | 29.163±5.214 |
| *E_SA_* | -3.843±0.71 | -2.887±0.658 |
| *G_binding energy_* | -19.166±4.469 | -14.592±3.016 |

*E_VDW_*: van der Waals energy

*E_ELE_*: eletrostatic energy

*E_GB_*: polar contribution to solvation

*E_SA_*: non-polar contribution to solvation

**Supplementary Figure 1** ECG reversed the alterations in the estrous cycle induced by ovariectomy (OVX) in mice. Representative images of vaginal crystal violet staining in mice before sacrificed (scale bar = 100 px)


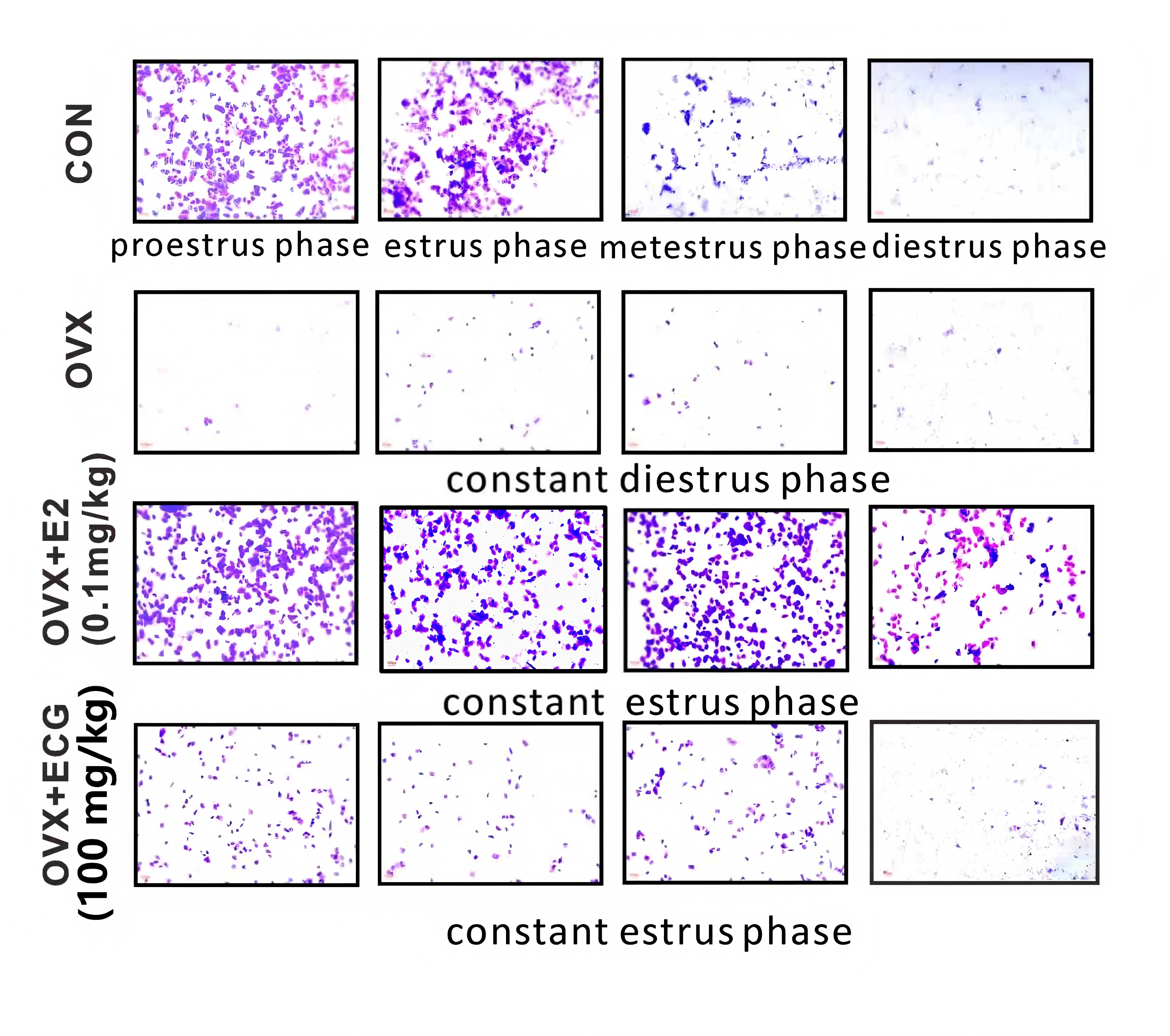

Supplement: Supplementary file 1 — Table S1: Primers used in qPCR analysis. TABLE S2: Compounds from ECG were identified by UHPLC‐Q‐Exactive analysis. TABLE S3: The binding energy by MMGBSA. Binding free energy components (kcal/mol) calculated by MM/GBSA for the complexes of ATK1 with SIN and NEO. FIGURE S1: ECG reversed the alterations in the estrous cycle induced by ovariectomy (OVX) in mice. Representative images of vaginal crystal violet staining in mice before sacrificed (scale bar = 100 px). [file FSN3-13-e71074-s001.docx]
